# Detection of miRNA in Cell Cultures by Using Microchip Electrophoresis with a Fluorescence-Labeled Riboprobe

**DOI:** 10.3390/s120607576

**Published:** 2012-06-07

**Authors:** Shohei Yamamura, Shouki Yatsushiro, Yuka Yamaguchi, Kaori Abe, Yasuo Shinohara, Masatoshi Kataoka

**Affiliations:** 1 Health Research Institute, National Institute of Advanced Industrial Science and Technology (AIST), 2217-14 Hayashi-cho, Takamatsu, Kagawa 761-0395, Japan; E-Mails: yamamura-s@aist.go.jp (S.Y.); yatsushiro.s@aist.go.jp (S.Y.); yukayama@suzuka-u.ac.jp (Y.Y.); k-abe@aist.go.jp (K.A.); 2 Division of Protein Expression, Institute for Genome Research, University of Tokushima, Kuramoto 3-18-15, Tokushima 770-8503, Japan; E-Mail: yshinoha@genome.tokushima-u.ac.jp

**Keywords:** microRNA, RNase protection assay, microchip electrophoresis

## Abstract

The analysis of a microRNA (miRNA), miR-222 isolated from the PC12 cell line, was performed by use of the ribonuclease (RNase) protection assay, cyanine 5 (Cy5)-labeled miR-222 riboprobe, and a Hitachi SV1210 microchip electrophoresis system, which can be used to evaluate the integrity of total RNA. The fluorescence intensity corresponding to the protected RNA fragment increased in a dose-dependent manner with respect to the complementary-strand RNA. More highly sensitive detection of miRNA by microchip electrophoresis than by conventional method using fluorescence-labeled riboprobe could be obtained in 180 s. An obvious increase in miR-222 expression induced by nerve growth factor in PC12 cells could be observed. These results clearly indicate the potential of microchip electrophoresis for the analysis of miRNA using RNase protection assay with a fluorescence-labeled riboprobe.

## 1. Introduction

The miRNAs are single-stranded RNA molecules of about 21∼23 nucleotides in length that are both ancient and highly conserved [1,2]. They serve as regulators of gene expression by modulating the translation and/or stability of messenger RNA targets and are crucial for the cellular changes that are necessary for cell development, tumorigenesis, apoptosis, and metabolism [3–7]. Several studies have implicated the aberrant expression of miRNAs in numerous diseases including some kinds of cancers and heart disease [8]. So, the importance of analysis for the expression of miRNAs is immensely increased in the field of not only cell biology but also that of clinical medicine.

Hybridization-based approaches, such as primer-extension, Northern blotting, miRNA microarray profiling, *in situ* hybridization, reverse-transcription PCR, and ribonuclease (RNase) protection are frequently used for the identification and quantification of known miRNAs [2]. The RNase protection assay is employed for the quantitative analysis of particular RNA expression in a heterogeneous RNA sample extracted from cells, and it can identify RNA molecules of known sequence even at a low concentration in a sample [9]. This method involves hybridization of test RNAs to complementary RNA probes (riboprobes), followed by digestion of the nonhybridized sequences with an RNase that specifically cleaves only single-stranded RNA but does not have activity against double-stranded RNA, *i.e.*, the hybridized RNA fragment. For analysis of hybridized RNA fragments, polyacrylamide gel or agarose gel electrophoresis is performed, followed by transfer to a membrane and measurement of the signal intensity of the labeled RNA [10,11]. Just like the RNase protection assay, the conventional methods are manual and time-consuming. Therefore, a more convenient, sensitive, and accurate method for the analysis of miRNA expression is desirable.

Microchip electrophoresis has received considerable interest in analytical chemistry due to its intrinsic characteristics of high speed, high throughput, low consumption of samples and reagents, miniaturization, and automation [12]. We recently reported that the RNase protection assay and cyanine (Cy5)-labeled riboprobe for the analysis of a particular mRNA in the total RNA extracted from cells can be performed by using a commercial instrument, the Hitachi SV1210 microchip electrophoresis system, which is used to evaluate the integrity of total RNA [9].

In the present study, we evaluated the ability of the Hitachi SV1210 to analyze miRNA expression in PC12 cells assessed by the RNase protection assay. The potential of microchip electrophoresis for the analysis of miRNA expression was shown, offering highly sensitive detection and ease of operation in a short time.

## 2. Experimental Section

### 2.1. Reagents

Dulbecco's modified Eagle's medium (DMEM), fetal calf serum (FCS), horse serum (HS), penicillin, and streptomycin were obtained from Life Technology, Co., (Carlsbad, CA, USA). Nerve growth factor (NGF) was obtained from Sigma-Aldrich Japan (Tokyo, Japan). SYBR Green II (concentration not given) was obtained from Molecular Probes, Inc. (Eugene, OR, USA). Dyna Marker RNA High and Low II (Biodynamics Laboratory Inc., Tokyo, Japan) were employed as a molecular weight marker for agarose gel electrophoresis and polyacrylamide gel electrophoresis, respectively.

### 2.2. PC12 Cell Cultures and RNA Preparation

The cell strain PC12 was obtained from the Japanese Collection of Research Bioresource Cell Bank. The cells were maintained in DMEM supplemented with 10% (v/v) FBS, 5% (v/v) HS, 100 unit/mL penicillin, and 100 μg/mL streptomycin as final concentrations in an atmosphere of 5% CO_2_ and 100% relative humidity at 37 °C. Cells were cultured according to the modified method described by Terasawa *et al.* [6]. For the isolation of RNA from NGF-activated PC12 cells, the cells were seeded onto culture dishes in a low concentration of serum (1% HS) for 24 h prior to treatment with 100 ng/mL NGF, and then differentiation was induced 48 h thereafter. For the isolation of RNA from non-activated PC 12 cells, the cells were seeded onto culture dishes containing medium with a low concentration of serum and no NGF.

Total RNA was extracted and purified from PC 12 cells with a Clontech Micro-Scale Total RNA Separator kit (Clontech Laboratories, Inc., Palo Alto, CA, USA). The concentration of purified total RNA was determined with a NanoDrop ND-100 apparatus (NanoDrop Technologies, Inc., Wilmington, DE, USA). For the evaluation of the integrity of the RNA, samples were separated by 1.0% agarose gel electrophoresis containing 13% formaldehyde, followed by SYBR Green II staining.

### 2.3. Synthesis of Riboprobe

The several types of the 21-bps synthetic oligonucleotides of miR-222 and 22-bps synthetic oligonucleotides of U 6 were purchased from Sigma-Aldrich Japan (Tokyo, Japan). The base sequences of each oligonucleotide and labeled types are shown in Table 1.

### 2.4. RNase Protection Assay

Twenty-microgram amounts of total RNA isolated from PC12 cell cultures or 0.039–40 ng of synthesized sense riboprobes, each with 10, 20, or 40 ng of Cy5-labeled riboprobe for microchip electrophoresis or 10, 20, or 40 ng of Cy3-labeled riboprobes for polyacrylamide gel electrophoresis, were used for the hybridization by use of an RPA III kit (Ambion Inc., Austin, TX, USA). The mixture was denatured at 98 °C for 3 min and subsequently hybridized at 80 °C for 1 h. After the hybridization, the single-stranded RNAs (non-protected RNA) were digested with RNase A/T1 (1/1,000 dilution) at 37 °C for 30 min. Thereafter the protected RNAs were purified and dissolved in 10 μL of ddw or 10 μL of sample buffer.

For the conventional RNase protection assay, the mixture of 2.0 μL of 6× formaldehyde gel-loading buffer and protected RNA sample with Cy3-miR-222 or Cy3-U6 riboprobes in 10 μL of ddw was denatured by heating at 65 °C for 15 min and standing on ice for 5 min. Samples were run on a denaturing polyacrylamide gel (12% polyacrylamide, 8 M urea) in 1× TAE buffer; and electrophoresis was performed at 250 V for 40 min, followed by staining with SYBR Green II. Thereafter the gels were scanned by using a BioRad Phoros FX system (BioRad Laboratories, Inc., CA, USA) for the quantitative analysis of fluorescence intensities.

For the RNase protection assay using microchip electrophoresis, the protected RNA samples with Cy5-miR-222 riboprobe or Cy5-U6 riboprobes in 10 μL of sample buffer were denatured as described above, and then subjected to microchip electrophoresis on the Hitachi SV1210 (Hitachi-Technologies Co., Ltd. Tokyo, Japan).

### 2.5. Microchip Preparation and Separation

**Figure 1.**
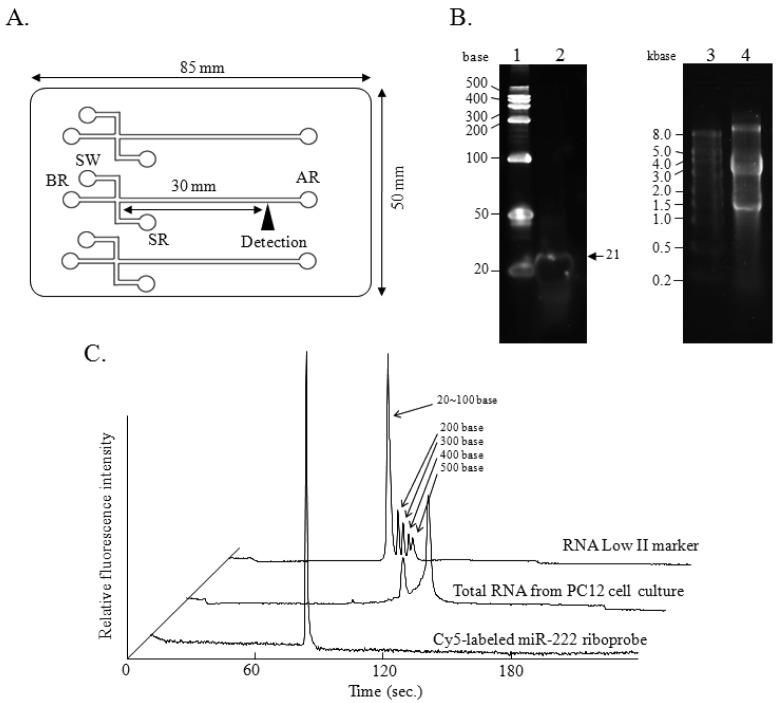
Design and size of the *i*-chip used for Hitachi SV1210 microchip electrophoresis. (**A**) Schematic diagram of the microchannel structure with cross-sections fabricated on a PMMA microchip, the i-chip (85 × 50 mm). The distances from each cross-section to the sample reservoir (SR), sample waste (SW), buffer reservoir (BR), and analysis reservoir (AR) well were 5.25, 5.25, 5.75, and 37.5 mm, respectively. (**B**) Separation of 1.0 μg of Cy5-labeled miR-22 riboprobe by 12% polyacrylamide gel electrophoresis (lane 2) and that of 10 μg of the total RNA (isolated from a PC12 cell culture) by 1.0% agarose gel electrophoresis (lane 4). RNA Low marker II (lane 1) and RNA High marker (lane 3) were used as sizing marker for each electrophoresis. (**C**) Electropherograms of 10 ng of the total RNA isolated from a PC12 cell culture and 100 fg of the Cy5-labeled miR-222 riboprobe.

A disposal *i*-chip 3 (Hitachi Chemical Co., Ltd., Tokyo, Japan), fabricated from polymethylmethacrylate (PMMA), and comprising an interconnected network of fluid reservoirs and microchannels, was used for all the experiments (Figure 1(A)). Three samples could be analyzed on one of these chips at one time. For analysis of total RNA, *i*-chips were prepared according to the manufacturer's instructions supplied with the *i*-RNA 12 kit (Hitachi Chemical Co., Ltd.), which included a gel containing a RNA-staining fluorescent dye. For the RNase protection assay or for analysis with the Cy5-labeled riboprobe, the *i*-RNA 12 kit was employed without the gel containing the RNA-staining dye because of the use of Cy5 for the detection of the protected RNA fragments. The loading gel was infused from the analysis reservoir (AR) well into the microchannels on the *i*-chip 3 by using a syringe, and the sample waste (SW) well and the buffer reservoir (BR) well were filled with 10 μL of gel by use of a pipette. RNA samples dissolved in 10 μL of sample buffer were added to the sample reservoir (SR).

Samples were loaded by electrokinetic injection, which was achieved by applying 300 V for 60 s to the SW well while grounding the other wells. The separation procedure was performed by applying a fixed 130 V to both the SR and SW wells, while the BR well was kept grounded. Simultaneously, 750 V was applied to the AR well to produce a suitable electric field in the separation channel. The semiconductor laser Charge Coupled Detector (CCD) detector (excitation at 635 nm and measurement of fluorescence at 660 nm) point was located 30 mm from the cross-section point. Each sample could be analyzed in parallel within 4 min.

### 2.6. Instrumentation

Experiments were performed by using a Hitachi SV1210 microchip electrophoresis instrument (Hitachi High-Technologies Co., Ltd.) equipped with a semiconductor laser CCD detector. The instrument consists of a bench-top device (chip reader) that is connected to a personal computer. The SV1210 software (version 1.6.1) includes data collection, presentation, and interpretation functions. The data are displayed as both simulated gel images and electropherograms. Electropherograms of the total RNA, *i.e.*, 18S and 28S ribosomal RNA fragments, are displayed in Figure 1(C).

## 3. Results and Discussion

### 3.1. Analysis of Cy5-miR-222 and Total RNA Isolated from a PC12 Cell Culture

As shown in Figure 1(B), the integrity of the Cy5-miR-222 riboprobe (lane 2, arrow) and that of the total RNA isolated from a PC12 cell culture (lane 4) were confirmed by denaturing polyacrylamide gel (12% polyacrylamide, 8 M urea) electrophoresis and 1.0% agarose gel electrophoresis (gel containing 13% formaldehyde), respectively, followed by SYBR Green II staining. By microchip electrophoresis, the integrity of the Cy5-miR-222 riboprobe was confirmed by using gel without the original dye for RNA staining, and the integrity of total RNA isolated from culture of PC12 was also confirmed by using the original gel containing the fluorescent dye for RNA staining (Figure 1(C)). Although a microgram quantity of RNA is needed for the examination of the integrity of RNAs by the conventional electrophoresis, only a nanogram amount of RNA is required in the microchip electrophoresis. Especially, a distinct single peak corresponding to the Cy5-miR-222 riboprobe was observed with just a 100 femtogram amount of RNA. Remarkably sensitive detection of the Cy5-labeled riboprobe was obtained, as the peak for the riboprobe was much higher than the peaks obtained for total RNA analysis with the original gel containing the RNA-staining dye. The semiconductor laser CCD detector of the Hitachi SV1210 (excitation at 635 nm and measurement of fluorescence at 670 nm) is used for the detection of RNA and was suitable for the detection of Cy5. Furthermore, the riboprobe was directly labeled with Cy5, so the background level was very low. These must contribute to the highly sensitive detection of the Cy5-labeled riboprobe without the use of the original gel containing RNA-staining dye. As shown in Figure 1(C), the single peak corresponding to the Cy5-labeled riboprobe was observed with a migration time of 79 s on the microchip electrophoretogram. To evaluate the reproducibility of the migration time in the different channels, we examined the total RNA in the original gel containing RNA-staining dye and Cy5-labeled riboprobe in the gel without the RNA-staining dye (Table 2). The relative standard deviations (RSD) for 5 different channels as to the migration time of 18S, 28S, and Cy5-labeled riboprobe were 0.36, 0.29, and 0.24%, respectively. These results indicate the reproducibility of the electrophoresis even in different channels with Cy5 labeling, and show that Cy5 was suitable for RNA labeling for the analysis by this microchip electrophoresis method.

### 3.2. miRNA Analysis Using Synthesized Riboprobes

**Figure 2.**
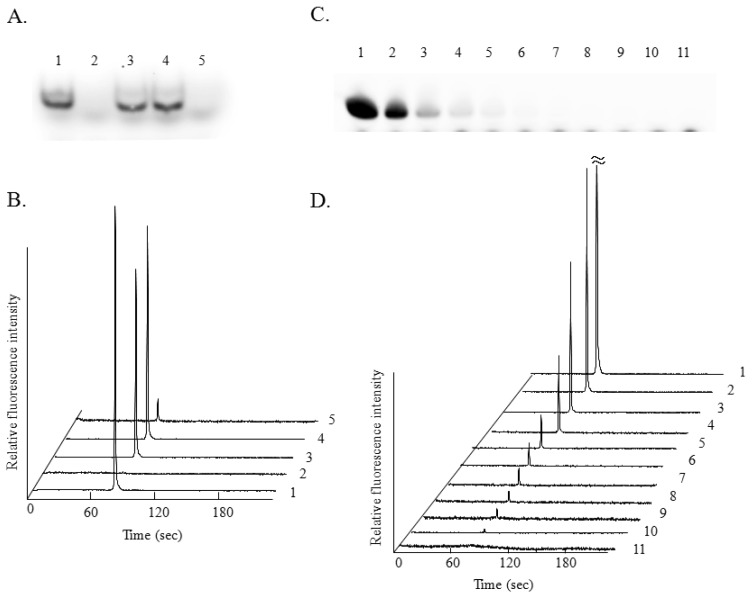
miRNA analysis using conventional electrophoresis and microchip electrophoresis. (**A**) Results of miRNA analysis using the conventional RNase protection assay with the Cy3-labeled miR-222 riboprobe. Lane 1: 10 ng of Cy3-labeled miR-222 riboprobe alone; lane 2: same probe with RNase treatment; lane 3: same probe hybridized with miR-222 sense riboprobe; lane 4: same probe hybridized with miR-222 sense riboprobe and subjected to RNase treatment and lane 5: same probe hybridized with miR-222 sense riboprobe in the presence of 100 ng of non-labeled miR-222 antisense riboprobe as a competitor and subjected to RNase treatment. (**B**) Electropherogram obtained upon analysis of 10 ng of the Cy5-labeled miR-222 riboprobe. Trace 1: Cy5-labeled miR-222 riboprobe alone; trace 2: same probe with RNase treatment; trace 3: same probe hybridized with miR-222 sense riboprobe; trace 4: same probe hybridized with miR-222 sense riboprobe and treated with RNase and trace 5: same probe hybridized with miR-222 sense riboprobe in the presence of 100 ng of non-labeled miR-222 antisense riboprobe as competitor and treated with RNase. (**C**) Conventional RNase protection assay using 40 ng of Cy3-labeled miR-222 antisense riboprobe with each of the indicated amounts of non-labeled miR-222 sense riboprobes (indicate correspondence between trace number and amount of non-labeled sense riboprobe: e.g., trace 1, 40 ng; trace 2, 20 ng; trace 3, 10 ng; trace 4, 5 ng; trace 5, 2.5 ng; trace 6, 1.25 ng; trace 7, 0.625 ng; trace 8, 0.313 ng; trace 9, 0.156 ng; trace 10, 0.078 ng; trace 11, 0.039 ng). (**D**) RNase protection assay using microchip electrophoresis with 40 ng of Cy5-labeled miR-222 antisense riboprobe and each of the indicated amounts (see “C”) of non-labeled miR-222 sense riboprobe.

miRNA analysis using 10 ng of Cy3-miR-222 riboprobe and 10 ng of the sense riboprobe for the conventional method using polyacrylamide gel was performed (Figure 2(A)). A single band corresponding to the Cy3-miR-222 riboprobe was observed (Figure 2(A), lane 1), and this band disappeared by RNase treatment (Figure 2(A), lane 2). By the hybridization of the Cy3-miR-222 riboprobe with the miR-222 sense riboprobe, single bands corresponding to the hybridized RNA fragment (double-stranded RNA) were observed with or without RNase treatment (Figure 2(A), lane 3 and 4). The band disappeared completely in the presence of 100 ng of non-labeled miR-222 as a competitor (Figure 2(A), lane 5). Cy3 was employed for antisense riboprobe labeling for use in the conventional gel electrophoresis, because we employed the BioRad Phoros FX system for gel scanning (excitation at 532 nm and measurement of fluorescence at 588 nm). miRNA analysis using 10 ng of the Cy5-miR-222 riboprobe and 10 ng of miR-222 sense riboprobe was then performed by using microchip electrophoresis (Figure 2(B)). A single peak corresponding to the Cy5-miR-222 riboprobe was observed (trace 1), and this peak completely disappeared by RNase treatment (trace 2). By the hybridization of the Cy5-miR-222 riboprobe with miR-222 sense riboprobe, single peaks corresponding to the hybridized RNA fragment and with similar fluorescent intensity were observed in the presence (trace 3) or absence (trace 4) of RNase. These results clearly indicate that protection of the double-stranded RNA from RNase digestion occurred when microchip electrophoresis using the Cy5-miR-222 riboprobe was performed, as in the case of the conventional RNase protection assay using the Cy3-miR-222 riboprobe for the analysis of miRNA. The peak corresponding to the Cy5-miR-222 riboprobe was observed at 79 s (trace 1), and single peaks corresponding to the hybridized RNA were observed at 81 s (trace 3 to 5). This difference in the migration time must be due to the slower migration of the double-stranded RNA fragment than that of the single-stranded one. In the presence of 100 ng of a competitor, the fluorescence intensity of the peak corresponding to the hybridized RNA was apparently lower than that in the absence of the competitor (trace 5). Although signal intensity corresponding to the hybridized RNA was hardly detectable by using the same amount of competitor for polyacrylamide gel electrophoresis (Figure 2(A), lane 5), significant fluorescent intensity could be detected by the microchip electrophoresis.

This difference may have been due to the more highly sensitive detection of Cy5-labeled RNA by microchip electrophoresis. The dose response of the test RNA (sense riboprobe) was examined by conventional miRNA analysis using polyacrylamide gel electrophoresis with the Cy3-miR-222 riboprobe (Figure 2(C)) and by microchip electrophoresis with the Cy5-miR-222 riboprobe (Figure 2(D)). The fluorescence intensity by each analysis increased in a sense riboprobe dose-dependent manner. Although a very weak band was observed in the hybridization with 5.0 ng of sense riboprobe in the conventional miRNA analysis (Figure 2(C), lane 4), an apparently single peak corresponding to the hybridized RNA was observed when 0.078 ng of the sense riboprobe was used for microchip electrophoresis (Figure 2(D), trace 10). These results indicate that miRNA analysis with the RNA protection assay using Cy5-antisense riboprobe and microchip electrophoresis afforded a highly sensitive detection.

In the present study, we employed Cy3 for riboprobe labeling in the conventional RNase protection assay for miRNA analysis. Digoxygenin (DIG) labeling of riboprobes for the RNase protection assay using the conventional method is frequently employed [13]. In an earlier study, we found the detection limit of RNase protection assay using the DIG-labeled riboprobe to be 5.0 ng [9]. The detection limit using fluorescence labeling in this study thus compared favorably with that using DIG labeling.

### 3.3. Increase in miR-222 Expression in PC12 Cells in Response to NGF Treatment

**Figure 3.**
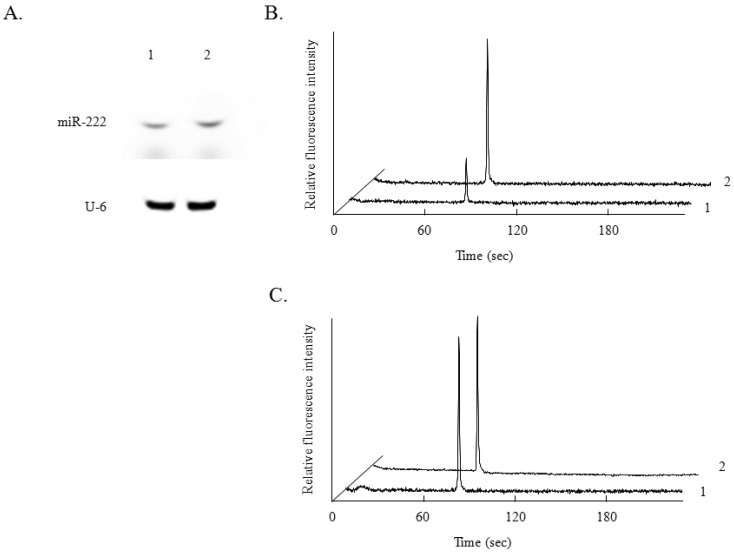
Analysis of miR-222 expression in PC12 cell cultures with (lane 2) or without (lane 1) NGF treatment by the conventional RNase protection assay (**A**) and by the RNase protection assay using microchip electrophoresis (**B**, **C**).

Terasawa *et al.* reported that expression of miR-222 is induced by NGF stimulation of PC12 cells, an established model of neuronal growth and differentiation, and that this expression contributes to NGF-dependent cell survival in the PC12 cell line [6]. By the conventional miRNA analysis, an obvious increase in miR-222 expression was observed after NGF treatment for 24 h (Figure 3(A)). On the other hand, U6 expression, as a control, was not affected by the NGF treatment. By miRNA analysis using microchip electrophoresis, a similar increase in the expression of miR-222 was observed (Figure 3(B)). The expression of U6 was also not changed by NGF treatment (Figure 3(C)), as in the case of the conventional method.

## 4. Conclusions/Outlook

We have shown the potential of microchip electrophoresis for rapid and highly sensitive analysis of miRNA expression in cells by using a Cy5-antisense riboprobe in the RNase protection assay. In previous work, expression of 248 bp mRNA was analyzed by using microchip electrophoresis with Cy5-labeled 248 bp antisense RNA probe [9]. Analysis of miRNA expression could be performed on microchip electrophoresis by using suitable length riboprobe, in this study. Analysis of miRNA expression is one of the most basic and frequently used procedures in cell biology and molecular biology. This application, *i.e.*, the analysis of miRNA expression by use of the RNase protection assay and microchip electrophoresis, will be useful for obtaining much information for a better understanding of cell development and functions.

## Figures and Tables

**Table 1.** Probe types for RNase protection assay.

**Probe type**	**Labeling**	**Sequence**
Cy5-miR-222 riboprobe	5′-Cy5	5′-ACCCAGUAGCCAGAUGUAGCU-3′ (21 bases)
Cy3-miR-222 riboprobe	5′-Cy3	5′-ACCCAGUAGCCAGAUGUAGCU-3′ (21 bases)
miR-222 competitor	none	5′-ACCCAGUAGCCAGAUGUAGCU-3′ (21 bases)
miR-222 sense riboprobe	none	5′-AGCUACAUCUGGCUACUGGGU-3′ (21 bases)
Cy5-U6 riboprobe	5′-Cy5	5′-CACGAAUUUGCGUGUCAUCCUU-3′ (22 bases)
Cy3-U6 riboprobe	5′-Cy3	5′-CACGAAUUUGCGUGUCAUCCUU-3′ (22 bases)
U6 competitor	none	5′-CACGAAUUUGCGUGUCAUCCUU-3′ (22 bases)
U6 sense riboprobe	none	5′-AAGGAUGACACGCAAAUUCGUG-3′ (22 bases)

**Table 2.** Reproducibility of migration times of 18S ribosomal RNA, 28S ribosomal RNA, and Cy5-labeled miR-222 riboprobe.

**Channel number**	**Total RNA**		**Cy5-miR-222 riboprobe**

	**18 S**	**28 S**	
1	108.5	121.6	79.1
2	107.6	120.9	79.3
3	108.1	121.2	78.8
4	108.2	121.2	78.9
5	108.6	121.8	79.0

Average (sec)	108.2	121.3	79.0
RSD (%)	0.36	0.29	0.24

## Abbreviations

AR: Analysis reservoir
BR: Buffer reservoir
CCD: Charge Coupled Device
Cy5: Cyanine dye
Cy3: Cyanine dye
ddw: double distilled water
DIG: Digoxygenin
DMEM: Dulbecco's modified Eagle's medium (DMEM)
FBS: Fetal bovine serum
HS: Horse serum
NGF: Nerve growth factor
miRNA: microRNA
PMMA: polymethylmethacrylate
Riboprobe: RNA probe
RNase: ribonuclease
SR: Sample reservoir
SW: Sample waste
TAE buffer: Tris/Tris-Acetate-EDTA buffer
